# Akt Inhibition Is Associated With Favorable Immune Profile Changes Within the Tumor Microenvironment of Hormone Receptor Positive, HER2 Negative Breast Cancer

**DOI:** 10.3389/fonc.2020.00968

**Published:** 2020-06-16

**Authors:** Douglas K. Marks, Robyn D. Gartrell, Margueritta El Asmar, Shuobo Boboila, Thomas Hart, Yan Lu, Qingfei Pan, Jiyang Yu, Hanina Hibshoosh, Hua Guo, Eleni Andreopoulou, Lisa Wiechmann, Katherine Crew, Joseph Sparano, Dawn Hershman, Eileen Connolly, Yvonne Saenger, Kevin Kalinsky

**Affiliations:** ^1^Perlmutter Cancer Center, NYU Langone Health, New York, NY, United States; ^2^Department of Pediatrics, Pediatric Hematology/Oncology and Medicine, Hematology/Oncology, Columbia University Irving Medical Center, New York, NY, United States; ^3^Bloomberg-Kimmel Institute for Cancer Immunotherapy, Johns Hopkins University School of Medicine, Baltimore, MD, United States; ^4^Herbert Irving Comprehensive Cancer Center, Columbia University, New York, NY, United States; ^5^College of Physicians and Surgeons, Columbia University Irving Medical Center, New York, NY, United States; ^6^Department of Computational Biology, St. Jude Children's Research Hospital, Memphis, TN, United States; ^7^Department of Pathology and Cell Biology, College of Physicians and Surgeons, Columbia University, New York, NY, United States; ^8^Weill Cornell Medicine, New York Presbyterian Hospital, New York, NY, United States; ^9^Department of Surgery, College of Physicians and Surgeons, Columbia University Irving Medical Center, New York, NY, United States; ^10^Department of Medicine, College of Physicians and Surgeons, Columbia University Irving Medical Center, New York, NY, United States; ^11^Department of Medicine, Albert Einstein College of Medicine, Montefiore Medical Center, Bronx, NY, United States; ^12^Department of Biostatistics, Mailman School of Public Health, Columbia University, New York, NY, United States; ^13^Division of Radiation Oncology, Columbia University Irving Medical Center, New York, NY, United States

**Keywords:** breast cancer, tumor microenvironment, MK-2206, AKT inhibitor, quantitative multiplex immunofluorescence, tumor immunobiology, pre-surgical

## Abstract

**Background:** The PI3K/Akt/mTOR pathway in part impacts tumorigenesis through modulation of host immune activity. To assess the effects of Akt inhibition on the tumor micro-environment (TME), we analyzed tumor tissue from patients with operable hormone receptor positive, HER2 negative breast cancer (BC) treated on a presurgical trial with the Akt inhibitor MK-2206.

**Methods:** Quantitative multiplex immunofluorescence (qmIF) was performed using CD3, CD8, CD4, FOXP3, CD68, and pancytokeratin on biopsy and surgical specimens of MK-2206 and untreated, control patients. nanoString was performed on surgical specimens to assess mRNA expression from MK-2206-treated vs. control patients.

**Results:** Increased CD3+CD8+ density was observed in post vs. pre-treatment tissue in the MK-2206-treated vs. control patients (87 vs. 0.2%, *p* < 0.05). MK-2206 was associated with greater expression of interferon signaling genes (e.g., *IFI6*, *p* < 0.05) and lower expression of myeloid genes (*CD163*, *p* < 0.05) on differential expression and gene set enrichment analyses. Greater expression of pro-apoptotic genes (e.g., *BAD*) were associated with MK-2206 treatment (*p* < 0.05).

**Conclusion:** Akt inhibition in operable BC was associated with a favorable immune profile in the TME, including increased CD3+CD8+ density and greater expression of interferon genes. Additional studies are warranted, as this may provide rationale for combining Akt inhibition with immunotherapy.

## Introduction

Targeted therapies have changed the treatment landscape of breast cancer (BC); however, until recently, BC was generally considered a minimally “immunogenic” malignancy and less likely to benefit from novel immunotherapeutic agents. While it has been established that lymphocyte rich tumors demonstrate increased chemosensitivity across BC subtypes, tumor infiltrating lymphocytes (TILs) have recently been identified as a candidate biomarker for efficacy of checkpoint inhibition (1–4). Of the BC subtypes, triple negative breast cancer (TNBC) has demonstrated an impressive response rate to immune checkpoint blockade combined with chemotherapy (2). By contrast, hormone receptor positive (HR+)/HER2− BC is frequently referred to as immunologically “cold” as these tumors have both lower TIL densities and response rates to immunotherapy as compared with TNBC (3, 5). There remains a significant need to identify effective strategies for augmenting the immunologic response in HR+/HER2− breast tumors, potentially including combination approaches (6–8).

The phosphatidylinositol 3 kinase (PI3K)/Akt murine thymoma viral oncogene (AKT)/mammalian target of rapamycin inhibitor (mTOR) pathway drives anti-apoptotic signaling and cell division in BC through activating point mutations, somatic copy number abnormalities, and increased gene expression (9). In addition to direct anti-neoplastic activity, there is growing evidence that agents targeting the PI3K/Akt/mTOR pathway have indirect anti-tumor activity mediated through the host immune response (10). While the PI3K/Akt/mTOR pathway is essential to immune cell maturation, the pathway also regulates expression of cytokines associated with recruitment of myeloid derived suppressor cells (MDSC) and regulatory T-cells as well as expression of PD-L1 on tumor cells (11). Specifically, treatment with the allosteric AKT inhibitor MK-2206 has previously demonstrated the capacity to downregulate PD-L1 at the transcriptional level in TNBC cell lines as well as augment the effect of a tumor specific vaccine in murine models (12, 13).

In this study, we define the impact of Akt inhibition on the tumor microenvironment (TME) in a series of patients with HR+/HER2− BC treated on a pre-surgical trial with MK-2206 (9). We performed *in situ* analysis with quantitative multiplex immunofluorescence (qmIF), on the pre-treatment core biopsies and post-treatment surgical specimens from patients treated with MK-2206 and evaluated differences in the TME compared to prospectively enrolled untreated controls. In addition, we performed transcriptomic expression analysis on the surgical specimens with nanoString to assess the effects of MK-2206 on the transcription of PI3K/Akt/mTOR pathway target genes as well as a broad panel of immune related genes.

## Methods

### Patient Samples

Archival tissue was collected from an open-label, single arm, presurgical trial with MK-2206 (NCT013195390). Patients were enrolled between October 2011 and March 2013 and received two weekly oral doses of MK-2206 prior to surgery: first dose at day−9 (+/− 1 day) and second dose at day−2 (+/− 1 day) from the date of surgery (9). Untreated control patients were prospectively accrued, and their tumor tissue was collected with the same methodology.

### qmIF

Four micrometer slides were stained using Opal^TM^ (Perkin Elmer, Hopkinton, MA) multiplex 6-plex kits for DAPI, CD3 (T-cells, LN10, Leica, 1:200), CD8 [Cytotoxic T-cells, 4B11, Leica, Ready to use (RTU)], CD68 (macrophages, KP1, 155 Biogenex, RTU), pancytokeratin (Tumor, PCK-26, Biocare, 1:200), CD4 (T helper cells, EPR6855, Abcam, 1:2000), and FOXP3 (T regulatory cells, 236A/E7, Abcam, 1:300). QmIF was performed on diagnostic core biopsies and surgical specimens by the recommended staining protocol including single stain controls and unstained controls. Five representative areas were selected to include three areas with tumor and up to 50% stroma and two areas with at least 90% tumor (Supplementary Figure 1). These images were factored equally into the analysis for each patient. For samples of small size, a minimum of two areas meeting the above criteria were required for inclusion. All images were confirmed as representative tumor areas by breast pathology (HH).

Images were captured using the Mantra^TM^ pathology workstation (PerkinElmer). Images were analyzed using inForm ^TM^ software (PerkinElmer) for tissue segmentation, cell segmentation, phenotyping, and scoring per previously published methods (Supplementary Figure 1B) (14). Cells were phenotyped for tumor, T-cells, macrophages, and other (negative for pancytokeratin, CD3, and CD68), then scored for concatenating variables CD4, CD8, and FOXP3. Subsequently, data obtained from all representative images were compiled to yield density values for each patient. Nearest neighbor analysis to assess for differences in spatial distribution of immune cell subsets following MK-2206 was performed by previously described methods (14). Image data was exported from inForm^TM^ version 2.2.1 (PerkinElmer). The inForm data from all images for each patient were processed in separate proprietary software designed in R Studio (version 0.99.896, Boston, MA). In this software, images were combined and analyzed to concatenate variables (i.e., CD3+CD4+FOXP3+) and determine density and distances of distinct phenotypes.

### NanoString

mRNA was manually extracted from FFPE slides of representative surgical specimens of patients who received MK-2206 (*n*= 5) or control (*n*= 5). Bioanalyzer calculations were performed to determine the quantity of mRNA (ng) to satisfy the quality requirements of nanoString platform. Subsequently, the PI3K (180 genes) and IO360 (770 genes) were run on these surgical specimens (15).

### Statistical Methods

For qmIF analysis, statistical analysis was performed using Mann-Whitney *U*-test. For nanoString analysis, data quality assessment, differential expression, and statistical comparison was performed by the systems biology data-driven network-based Bayesian inference of drivers (NetBID, https://github.com/jyyulab/NetBID) (16). Gene Set Enrichment Analysis (GSEA) was performed using MSigDB (v6.1) and “fgsea” package in R, with default parameters. Multiple comparison analysis was performed using Benjamini-Hochberg method.

### Ethics

Patient provided written consent to participate in the presurgical study which was approved by the Columbia University Irving Medical Center and Albert Einstein Cancer Center institutional review boards.

## Results

### Patient Population

Between September 2011 and July 2014, 12 patients with newly diagnosed invasive BC were prospectively enrolled to receive MK-2206 (9). In addition, tissue from diagnostic core biopsies and surgical specimens were prospectively collected on 6 untreated controls (9). For this analysis, seven patients treated with MK-2206 and one control patient were excluded for insufficient tissue. One MK-2206-treated patient was excluded from qmIF analysis due to poor tissue quality. In total, 9 patients with paired biopsy and surgical specimens (5 MK-2206 and 4 control) had evaluable tissue for qmIF analysis. 10 surgical specimens (5 MK-2206 and 5 control) were evaluable for nanoString.

All patients included in our analyses had HR+/HER2− tumors with invasive ductal histology. Of the MK-2206-treated patients included in this study, one patient received the 200 mg dose, one patient 135 mg, and the remaining three with 90 mg (Supplementary Table 1 for clinicopathologic features).

### Treatment With MK-2206 Increases CD3+CD8+ Cytotoxic T-Cell (CTL) Density

**Figure 1:**
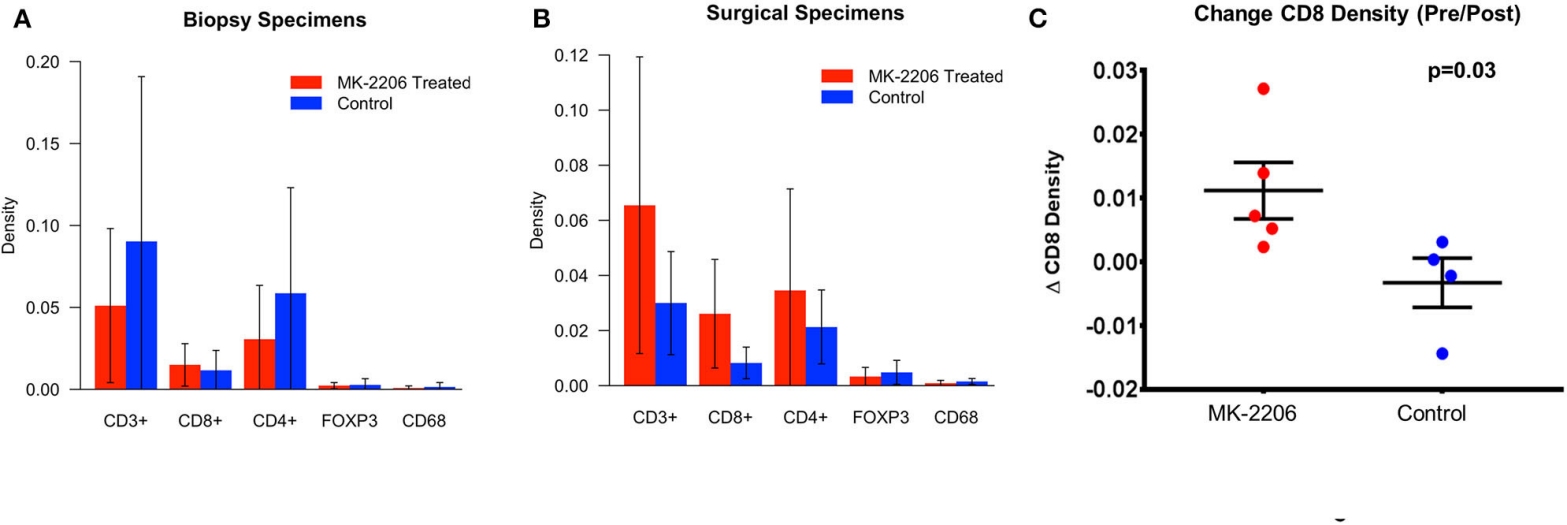
qMIF Analysis of biopsy and surgical specimens from a pre-surgical trial with MK-2206. **(A)** Mean density by immune cell subtype in biopsy specimens of MK-2206 (red) vs. control (blue). **(B)** Mean density by immune cell subtype in surgical specimens of MK-2206 (red) vs. control (blue). **(C)** Change in CD8 density between baseline biopsy and surgical specimen of MK-2206-treated (red) and control (blue) patients.

The density of each immune subset was measured by qMIF analysis at the time of biopsy and in the surgical specimen (Figures 1A,B). As frequently seen in HR+/HER2− BC, the baseline immune infiltrate was observed to be modest in the biopsy specimens from the MK-2206 and control groups, with lymphocytes representing the majority of the immune infiltrate in this cohort (17). Patients treated with MK-2206 exhibited a significant increase in median cytotoxic T-cells (CD3+CD8+) density, as compared to untreated control patients for whom no change was observed (87 vs. 0.2%, *p*= 0.03, Figure 1C, Supplementary Figures 2–3). No change was detected in the macrophage (CD68), T helper (CD4) T reg (CD4+FOXP3+) density following MK-2206 treatment as compared to paired pathology specimens from controls. A numerical increase in the CD8/FOXP3 ratio was observed in MK-2206-treated patients, which demonstrated a higher mean CD8/FOXP3 ratio in post treatment specimens as compared to control patients (19.4 vs. 4.6), although this finding did not reach statistical significance (*p*= 0.32).

Using nearest neighbor analysis, we observed a numerical reduction in median pixel distance (−12.5%) between CTL cells and tumor cells following treatment, suggesting that the increased density of effector T-cells is not relegated to the periphery. This observation was not seen in the control group when comparing the baseline biopsy to surgical excision specimen.

### MK-2206 Associated With Gene Expression Change in Downstream Targets of PI3K/Akt/mTOR Pathway

mRNA expression analysis confirms the *in-vivo* inhibitory activity of MK-2206 on PI3K/AKT/mTOR pathway. MK-2206 was associated with lower mean expression levels of genes associated with cell cycle progression including *CTNNB1* (raw *p*= 0.01) and *CCND2* (raw *p*= 0.02) in comparing post-surgical specimens from MK-2206-treated patients to control. Additionally, greater expression of pro-apoptotic gene products *BAD* (raw *p* < 0.01) *DDIT* (raw *p*= 0.03) was observed in surgical specimens after MK-2206 vs control. Consistent, with these findings, a trend toward greater *CASP9* expression was also found (raw *p*= 0.067). No significant difference in expression of *BAX* was observed between the two groups.

**Figure 2:**
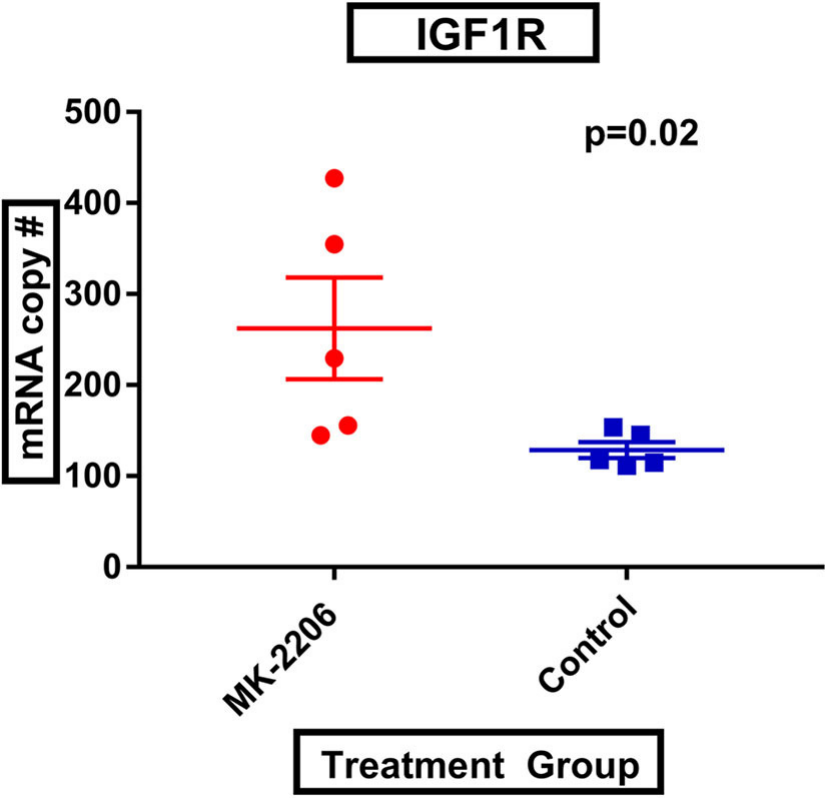
MK-2206 inhibition associated with greater expression of IGF-1R. IGF-1R expression was found to be significantly higher in the surgical specimens from the MK-2206-treated (red) vs. control (blue) patients (*p* = 0.02).

Following MK-2206, mean *IGF-1R* expression was greater in MK-2206-treated patients as compared to untreated controls (472.4 vs. 226.0 copies, raw *p* = 0.01) (Figure 2). We also observed greater expression of *HER3* with double the mean mRNA copy number for *HER3*; although, the difference did not reach statistical significance (1060.6 vs. 577.4 copies, raw *p*= 0.14).

### Transcriptomic Analysis Highlights Greater Expression of Interferon Related Gene Expression and Lower Expression of Myeloid Related Genes in MK-2206-Treated Patients

**Figure 3:**
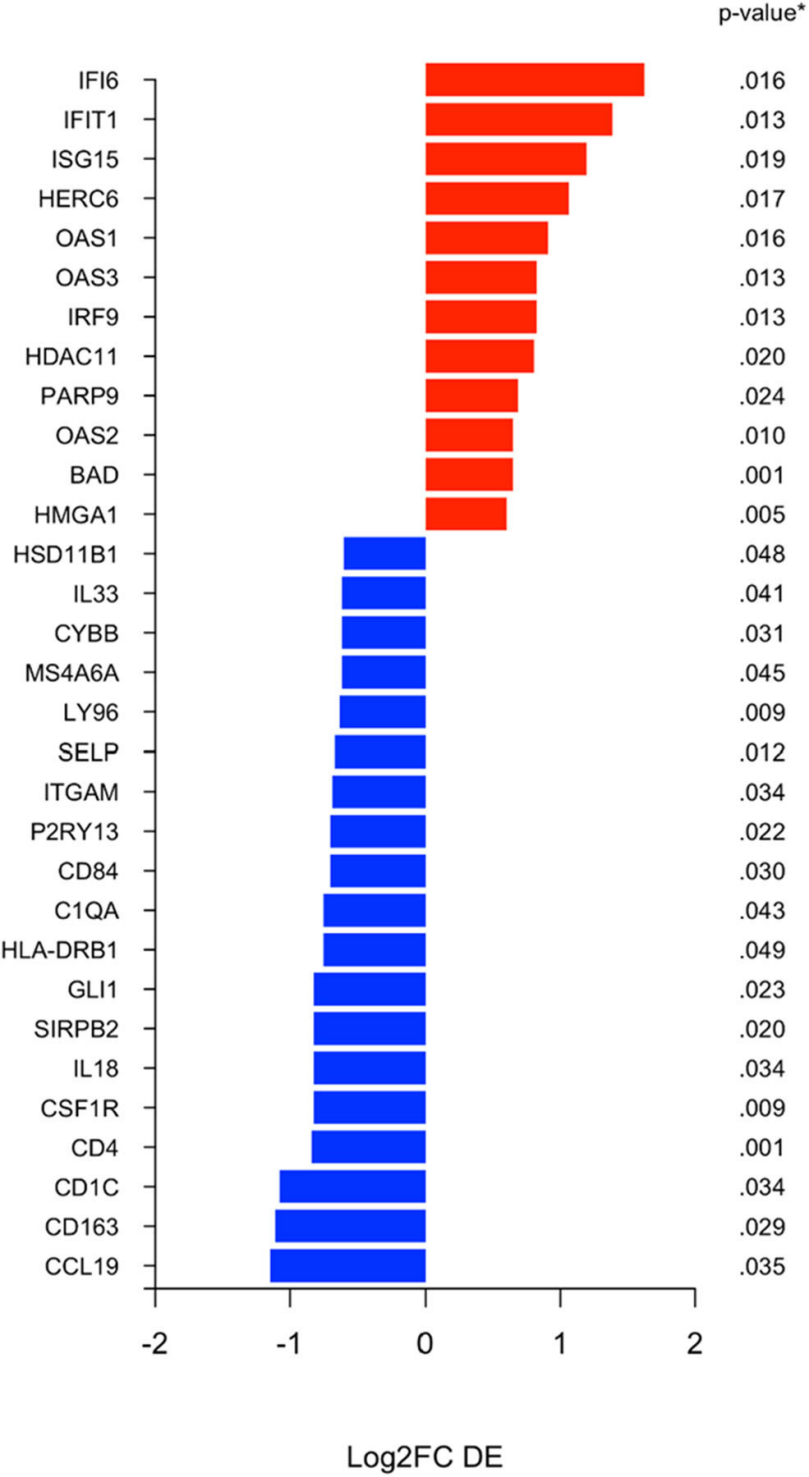
Differential expression (DE) analysis of immune related genes. Differential analysis identified selection genes as either 1.5 fold increased (black) or 1.5 fold decreased (red) in the surgical specimens after MK-2206, using untreated surgical samples (control) as reference baseline.

Using the nanoString 770 gene IO-360 panel, differential expression (DE) between surgical specimens from patients treated with MK-2206 vs. untreated controls identified 31 genes with 1.5-fold higher/lower expression (Figure 3) (15). Mean expression levels of myeloid related genes, including *CD163* (raw *p*= 0.03), *CSF1R* (raw *p* < 0.01), *HLA-DR* (raw *p*= 0.05), *P2RY13* (raw *p*= 0.02), *ITGAM* (raw *p*= 0.03), *MS4A6A* (raw *p*= 0.04), were lower in the surgical specimens from patients treated with MK-2206 compared to control.

By contrast, of the immune genes with greater expression in the MK-2206 patients, the majority were related to interferon related signaling and included *IFI6* (*p* = 0.02), *IFIT1* (*p* = 0.01), *ISG15* (*p* = 0.02), *OAS1* (*p* = 0.02), *IRF9* (*p* = 0.01), and *OAS2* (*p* = 0.01) (Figure 3).

**Figure 4:**
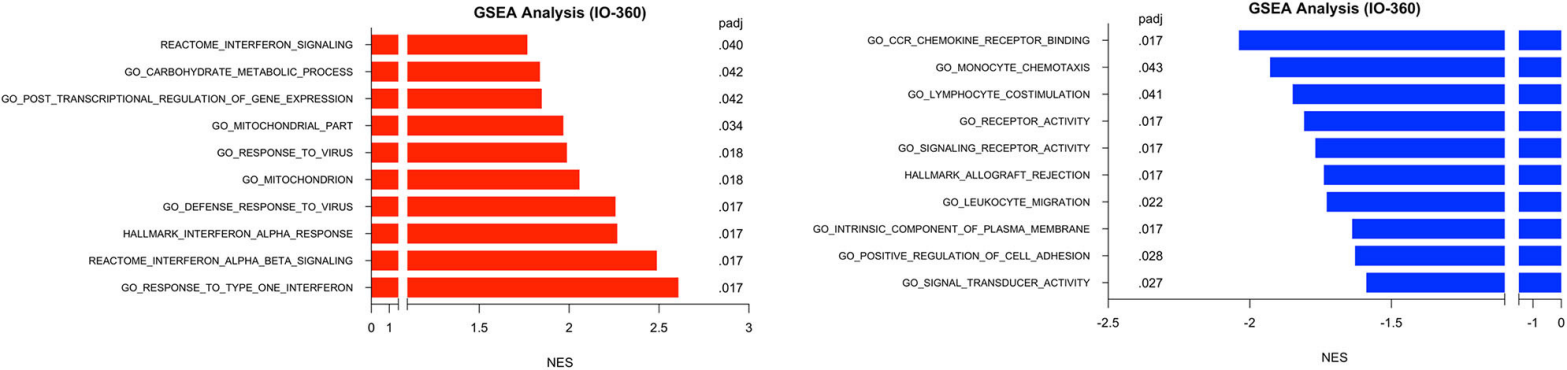
Gene set enrichment analysis (GSEA) for Immune Related Genes. GSEA was performed using pathways derived from gene sets from Molecular Signatures Database (GO, HALLMARK, REACTOME gene sets). Plot above depicts top 10 pathways observed to be at least 1.5 fold increased (black) or 1.5 decreased (red) from surgical samples from MK-2206-treated patients, using untreated patients as a reference baseline which were found to be statistically significant by multiple comparison analysis.

GSEA was performed in surgical samples with the untreated group considered the reference baseline, and 23 pathways were found to be enriched, with 10 pathways increased and 13 pathways decreased. Three distinct canonical gene sets ascribed to interferon signaling, *GO, HALLMARK and REACTOME* were observed to be statistically increased in the MK-2206-treated surgical samples by multiple comparisons (all raw *p* < 0.001, adj *p* < 0.02), whereas genes ascribed to monocyte chemotaxis were decreased (raw *p* < 0.01, adj *p* = 0.04) (Figure 4) (18). As expected, gene sets highlighting carbohydrate metabolic processing (raw *p* < 0.001, adj *p* = 0.04) were increased in the post-MK-2206 samples.

## Discussion

To our knowledge, this is the first study to perform a comprehensive evaluation of the TME in patient breast tumor samples following treatment with an AKT inhibitor. After only two doses, in the window-of-opportunity study, our findings support direct and indirect activity of AKT inhibition on the TME.

Consistent with previous preclinical observations in BC cell lines, and reflecting known direct anti-neoplastic activity of AKT inhibition, we observed greater expression of pro-apoptotic genes and upstream receptor tyrosine kinases including *IGF-1R* and *HER3* following treatment with MK-2206 (19). Additionally, we identified higher levels of *GSK3A* (raw *p*= 0.02), a critical negative regulator of glycogen synthase, in the surgical samples from MK-2206-treated patients as compared to untreated controls, which is consistent with the important role of the PI3K pathway in metabolic regulation (10). These findings provide a transcriptomic basis for the increased serum glucose level (*p* = 0.02), insulin (*p* < 0.01) and C-peptide (*p* < 0.01) levels previously reported in the MK-2206 pre-surgical trial (9).

Notably, our data provides support from a clinical trial that is consistent with pre-clinical findings that that inhibition of the PI3K/AKT pathway are capable of increasing CD8 density in mouse models (13, 20). Therapeutics capable of augmenting CTL density within the TME would be expected to promote a more effective anti-tumor immune response, as high baseline CTL (CD8+) density are associated with chemosensitivity across BC subtypes and improved survival in TNBC and HER2 amplified BC (3, 21). In accordance with the increase in CD8 density by qMIF, we identified a higher expression of single interferon genes, as well as, gene sets related the interferon using the nanoString platform in MK-2206 post-treatment surgical samples. These signatures positively regulate T lymphocyte cytotoxicity and have been found to be upregulated in the context of other locoregional techniques which have demonstrated synergy with immunotherapy (6).

In parallel to our findings regarding CTL activity, we observed decreased expression of myeloid genes, which may contribute to the biologic mechanism underlying the increase in CD8 density and greater expression of cytotoxicity immune signatures seen following MK-2206. In our analysis, Akt inhibition was associated with lower expression levels of both individual myeloid related genes as well as several canonical myeloid gene sets on GSEA. Our observation is consistent with preclinical co-culture experiments with THP-1 cells and MCF7 breast cancer cells which support the PI3K pathway having a critical role in the modulation of macrophage activity in the TME which has in turn been implicated in impaired CTL response and increased metastatic potential (22). The clinical relevance of these findings are supported by clinicopathologic studies in human BC which have demonstrated that increased baseline myeloid cell density as well as expression of myeloid genes, including *CD163*, are associated with worse BC outcomes (22, 23).

## Study Limitations and Future Directions

These studies were performed on specimens from a trial that was terminated early due to grade III rash, mucositis, and pruritus, limiting the sample size available for evaluation. Additionally, while we intended to perform transcriptomic analysis on both the pre-treatment and post-treatment tissues specimens, as was done with qMIF, we were only able to perform this analysis on the tissue obtained from surgical resection due to inadequate RNA quantity from biopsy specimens. Despite performing multiplex approaches, B-lymphocyte, natural killer cell, and MDSC activity were underrepresented in our analysis. Preclinical data indicate that these immune subsets likely play an important role in the TME in BC and warrant investigation in further studies (24).

Lastly, equivalent effect on the TME cannot be assumed for agents that target alternative AKT isoforms or other components of the PI3K pathway and may not be generalizable for hormone receptor negative or HER2 amplified BC, as differences in immunobiology exist between BC subtypes (3).

## Summary

As preclinical data exist to supports activity of PI3K targeted agents on the immune microenvironment, the goal of this study was to assess the impact of Akt inhibition on the TME of HR+/HER2− BC in samples collected in a pre-surgical clinical trial. By qmIF and targeted genomic expression analysis, we confirmed in human breast cancer specimens the biologic activity of MK-2206, with well-described changes in gene expression of known targets of the PI3K/Akt/mTOR pathway as well as marked indirect immune related effects of Akt inhibition on the TME including an increase in CTL density as well as greater expression of interferon related genes and lower expression of myeloid genes.

In BC, particularly in tumors expressing the estrogen receptor, benefit from immunotherapeutic approaches has been modest and alternative strategies to augment host immune response are needed. Increased expression of interferon signatures is associated with improved relapse-free survival in BC patients, prompting development of agents aimed at specifically increasing CTL infiltration and interferon signaling with efficacy signals observed in other tumor types (25). Our findings support that agents targeting the PI3K pathway lead to a favorable change in the immune microenvironment and provide rationale for combining these agents with immunotherapeutics.

## Data Availability Statement

Publicly available datasets were analyzed in this study. This data can be found here: the NCBI Gene Expression Omnibus (GSE150512).

## Ethics Statement

Patient provided written consent to participate in the presurgical study which was approved by the Columbia University Irving Medical Center and Albert Einstein Cancer Center institutional review boards.

## Author Contributions

DM and RG contributed to study design, performed qMIF and nanostring analyses, final data analysis, preparation of manuscript and provided study supervision. ME performed qMIF and nanostring analyses, final data analysis, preparation of manuscript. SB contributed to final data analysis, preparation of manuscript. TH, QP, and JY contributed to final data analysis. YL performed qMIF and nanostring analyses. HH and HG: reviewed pathology specimens and qMIF images, preparation of manuscript. KC, JS, and DH contributed to study design. EC, YS, and KK contributed to the study design, final data analysis, preparation of manuscript, and provided study supervision. All authors contributed to the article and approved the submitted version.

## Conflict of Interest

KK has consultancy or advisory positions with bioTheranostics, Lilly, Pfizer, Amgen, Novartis, Eisai, AstraZeneca, Ipsen, Genentech/Roche, Immunomedics, Odonate Therapeutics and immediate family member(s) with stock/ownership interest in Novartis and Array Biofarma. KK has also received fees to speak on behalf of Lilly. JY reports having received consultancy fees from Mount Sinai Health System. The remaining authors declare that the research was conducted in the absence of any commercial or financial relationships that could be construed as a potential conflict of interest.

## Supplementary Material

Supplementary Table 1 Treatment and demographic data from MK-2206 and untreated control cohorts.

Supplementary Figure 1 Change in CTL (C3+CD8+) density between diagnostic biopsies and surgical specimens in MK-2206 treated patients vs. control. M = MK-2206 treated, C = untreated. MK-2206 dose, M1: 200 mg dose, M2: 135 mg, M3: 90 mg, M4: 90 mg, M5: 90 mg. ^*^PI3KCA mutation. ^**^PTEN mutation.

Supplementary Figure 2 Representative qMIF image of pretreatment and surgical specimen of MK-2206 treated patient. **(A)** qmIF image from pretreatment biopsy of patient selected to receive MK-2206. **(B)** qmIF image of post treatment pathology for same patient. Blue (Dapi/Nuclear), Red (Pancytokeratin), Cyan (CD3+), Magenta (CD8+), Yellow (FOXP3).

Supplementary Figure 3 qmIF images demonstrating multiplex staining. **(A)** Dapi/Nuclear single stain (blue) **(B)** Dapi + pancytokeratin (red) multiplex image, red dot represents training program for cell phenotype (cell assigned as carcinoma based on pancytokeratin staining and nuclear features) **(C)** Dapi (blue), pancytokeratin (red) and CD3 (cyan) multiplex image **(D)** pancytokeratin (red), CD8 (magenta), and FOXP3 (yellow) multiplex image **(E)** Dapi (blue), pancytokeratin (red), CD4 (orange), CD68 (green) multiplex image.

## References

[1] Loi S Adams S Schmid P Cortés J Cescon DW Winer EP LBA13Relationship between tumor infiltrating lymphocyte (TIL) levels and response to pembrolizumab (pembro) in metastatic triple-negative breast cancer (mTNBC): results from KEYNOTE-086. Ann Oncol. (2017) 28:mdx440005-mdx440.005. 10.1093/annonc/mdx440.005

[2] Schmid P Adams S Rugo HS Schneeweiss A Barrios CH Iwata H. Atezolizumab and Nab-Paclitaxel in advanced triple-Negative breast cancer. N Engl J Med. (2018) 379:2108–21. 10.1056/NEJMoa1809615 30345906

[3] Denkert C von Minckwitz G Darb-Esfahani S Lederer B Heppner BI Weber KE. Tumour-infiltrating lymphocytes and prognosis in different subtypes of breast cancer: a pooled analysis of 3771 patients treated with neoadjuvant therapy. Lancet Oncol. (2018) 19:40–50. 10.1016/S1470-2045(17)30904-X 29233559

[4] Loi S Drubay D Adams S Pruneri G Francis PA Lacroix-Triki M. Tumor-Infiltrating lymphocytes and prognosis: a Pooled individual patient analysis of early-Stage triple-Negative breast cancers. J Clin Oncol. (2019) 37:559–69. 10.1200/JCO.18.01010 30650045 PMC7010425

[5] Vonderheide RH LoRusso PM Khalil M Gartner EM Khaira D Soulieres D. Tremelimumab in combination with exemestane in patients with advanced breast cancer and treatment-associated modulation of inducible costimulator expression on patient t cells. Clin Cancer Res. (2010) 16:3485–94. 10.1158/1078-0432.CCR-10-0505 20479064

[6] McArthur HL Diab A Page DB Yuan J Solomon SB Sacchini V. A pilot study of preoperative single-Dose ipilimumab and/or cryoablation in women with early-Stage breast cancer with comprehensive immune profiling. Clin Cancer Res. (2016) 22:5729–37. 10.1158/1078-0432.CCR-16-0190 27566765 PMC5161031

[7] Isakoff SJ Tolaney SM Tung NM Adams S Soliman HH Brachtel EF A phase 1b study of safety and immune response to PVX-410 vaccine alone and in combination with durvalumab (MEDI4736) in HLA-A2+ patients following adjuvant therapy for stage 2/3 triple negative breast cancer. J Clin Oncol. (2017) 35:TPS1126-TPS1126. 10.1200/JCO.2017.35.15_suppl.TPS1126

[8] Nanda R Liu MC Yau C Pembrolizumab plus standard neoadjuvant therapy for high-risk breast cancer (BC): results from I-SPY 2. J Clin Oncol. (2017) 35:506 10.1200/JCO.2017.35.15_suppl.50628029304

[9] Kalinsky K Sparano JA Zhong X Andreopoulou E Taback B Wiechmann L. Pre-surgical trial of the AKT inhibitor MK-2206 in patients with operable invasive breast cancer: a new york cancer consortium trial. Clin Transl Oncol. (2018) 20:1474–83. 10.1007/s12094-018-1888-2 29736694 PMC6222014

[10] Okkenhaug K Graupera M Vanhaesebroeck B. Targeting PI3K in cancer: impact on tumor cells, their protective stroma, angiogenesis, and immunotherapy. Cancer Discov. (2016) 6:1090–105. 10.1158/2159-8290.CD-16-0716 27655435 PMC5293166

[11] Xue G Zippelius A Wicki A Mandalà M Tang F Massi D. Integrated Akt/PKB signaling in immunomodulation and its potential role in cancer immunotherapy. J Natl Cancer Inst. (2015) 107:djv171. 10.1093/jnci/djv171 26071042

[12] Mittendorf EA Philips AV Meric-Bernstam F Qiao N Wu Y Harrington S. PD-L1 expression in triple-negative breast cancer. Cancer Immunol Res. (2014) 2:361–70. 10.1158/2326-6066.CIR-13-0127 24764583 PMC4000553

[13] Abu-Eid R Samara RN Ozbun L Abdalla MY Berzofsky JA Friedman KM. Selective inhibition of regulatory t cells by targeting the PI3K-Akt pathway. Cancer Immunol Res. (2014) 2:1080–9. 10.1158/2326-6066.CIR-14-0095 25080445 PMC4221428

[14] Gartrell RD Marks DK Hart TD Li G Davari DR Wu A. Quantitative analysis of immune infiltrates in primary melanoma. Cancer Immunol Res. (2018) 6:481–93. 10.1158/2326-6066.CIR-17-0360 29467127 PMC5882545

[15] Cesano A Warren S. Bringing the next generation of immuno-oncology biomarkers to the clinic. Biomedicines. (2018) 6:14. 10.3390/biomedicines6010014 29393888 PMC5874671

[16] Du X Wen J Wang Y Karmaus PWF Khatamian A Tan H. Hippo/Mst signalling couples metabolic state and immune function of CD8α+ dendritic cells. Nature. (2018) 558:141–5. 10.1038/s41586-018-0177-0 29849151 PMC6292204

[17] Burugu S Asleh-Aburaya K Nielsen TO. Immune infiltrates in the breast cancer microenvironment: detection, characterization and clinical implication. Breast Cancer. (2017) 24:3–15. 10.1007/s12282-016-0698-z 27138387

[18] Subramanian A Tamayo P Mootha VK Mukherjee S Ebert BL Gillette MA. Gene set enrichment analysis: a knowledge-based approach for interpreting genome-wide expression profiles. Proc Natl Acad Sci U S A. (2005) 102:15545–50. 10.1073/pnas.0506580102 16199517 PMC1239896

[19] Chandarlapaty S Sawai A Scaltriti M Rodrik-Outmezguine V Grbovic-Huezo O. AKT inhibition relieves feedback suppression of receptor tyrosine kinase expression and activity. Cancer Cell. (2011) 19:58–71. 10.1016/j.ccr.2010.10.031 21215704 PMC3025058

[20] Peng W Chen JQ Liu C Malu S Creasy C Tetzlaff MT. Loss of PTEN promotes resistance to t Cell-Mediated immunotherapy. Cancer Discov. (2016) 6:202–16. 10.1158/1538-7445.AM2016-4363 26645196 PMC4744499

[21] Ali HR Provenzano E Dawson SJ Blows FM Liu B Shah M. Association between CD8+ t-cell infiltration and breast cancer survival in 12,439 patients. Ann Oncol. (2014) 25:1536–43. 10.1093/annonc/mdu191 24915873

[22] Vergadi E Ieronymaki E Lyroni K Vaporidi K Tsatsanis C. Akt signaling pathway in macrophage activation and M1/M2 polarization. J Immunol. (2017) 198:1006–14. 10.4049/jimmunol.1601515 28115590

[23] Medrek C Ponten F Jirstrom K Leandersson K. The presence of tumor associated macrophages in tumor stroma as a prognostic marker for breast cancer patients. BMC Cancer. (2012) 12:306. 10.1186/1471-2407-12-306 22824040 PMC3414782

[24] Brown JR Wimberly H Lannin DR Nixon C Rimm DL Bossuyt V. Multiplexed quantitative analysis of CD3, CD8, and CD20 predicts response to neoadjuvant chemotherapy in breast cancer. Clin Cancer Res. (2014) 20:5995–6005. 10.1158/1078-0432.CCR-14-1622 25255793 PMC4252785

[25] Ribas A Dummer R Puzanov I VanderWalde A Andtbacka RHI Michielin O. Oncolytic virotherapy promotes intratumoral t Cell infiltration and improves anti-PD-1 immunotherapy. Cell. (2017) 170:1109–1119.e10. 10.1016/j.cell.2017.08.027 28886381 PMC8034392

