# Supplementary figures and images for: Akt Inhibition Is Associated With Favorable Immune Profile Changes Within the Tumor Microenvironment of Hormone Receptor Positive, HER2 Negative Breast Cancer
Source: Front Oncol. 2020 Jun 16;10:968. doi: 10.3389/fonc.2020.00968 (PMC7308467; doi:10.3389/fonc.2020.00968)

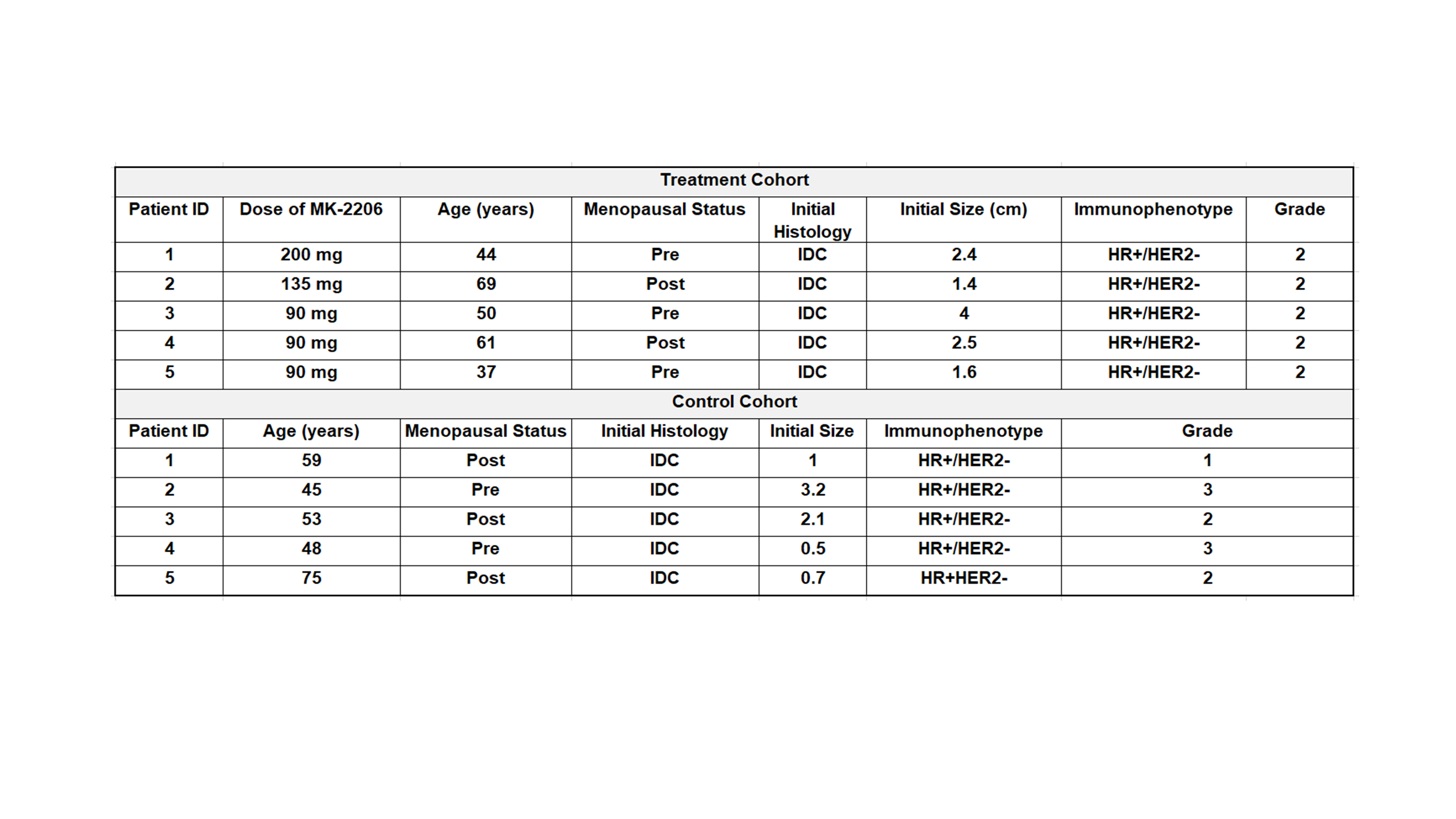

Supplement: Supplementary Table 1 — Treatment and demographic data from MK-2206 and untreated control cohorts. [file Image_1.TIF]

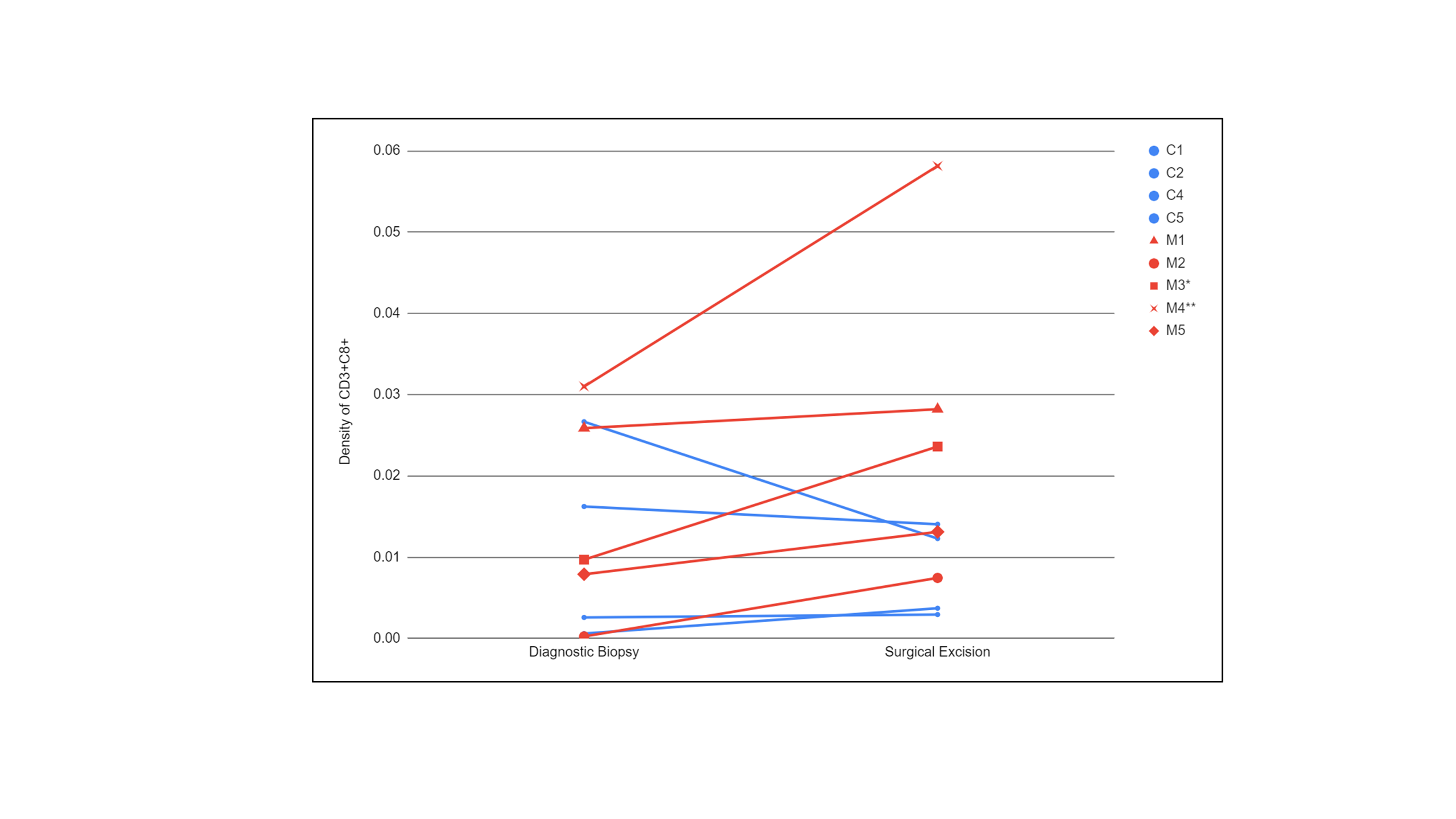

Supplement: Supplementary Figure 1 — Change in CTL (C3+CD8+) density between diagnostic biopsies and surgical specimens in MK-2206 treated patients vs. control. M = MK-2206 treated, C = untreated. MK-2206 dose, M1: 200 mg dose, M2: 135 mg, M3: 90 mg, M4: 90 mg, M5: 90 mg. *PI3KCA mutation. **PTEN mutation. [file Image_2.TIF]

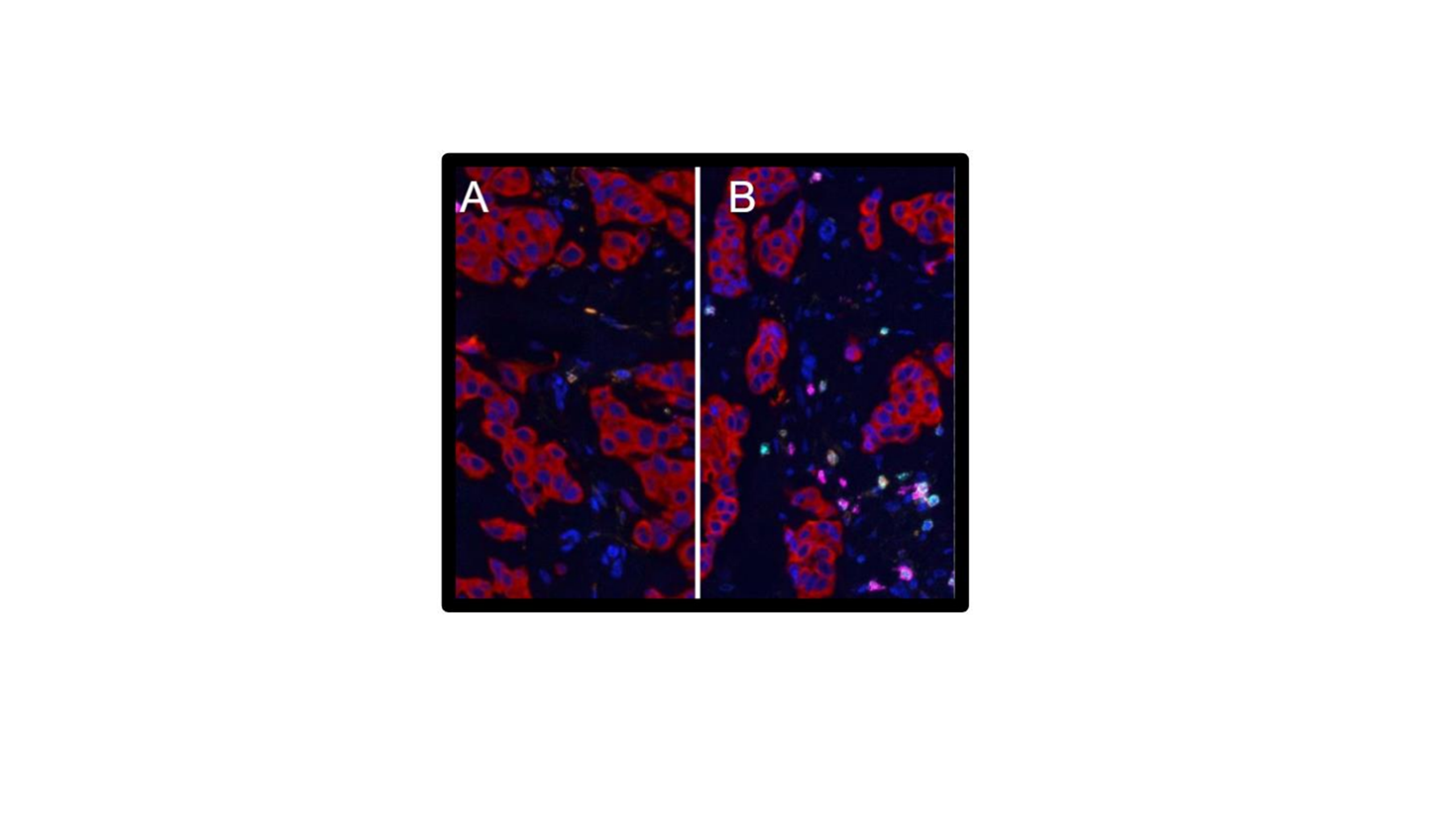

Supplement: Supplementary Figure 2 — Representative qMIF image of pretreatment and surgical specimen of MK-2206 treated patient. (A) qmIF image from pretreatment biopsy of patient selected to receive MK-2206. (B) qmIF image of post treatment pathology for same patient. Blue (Dapi/Nuclear), Red (Pancytokeratin), Cyan (CD3+), Magenta (CD8+), Yellow (FOXP3). [file Image_3.TIF]

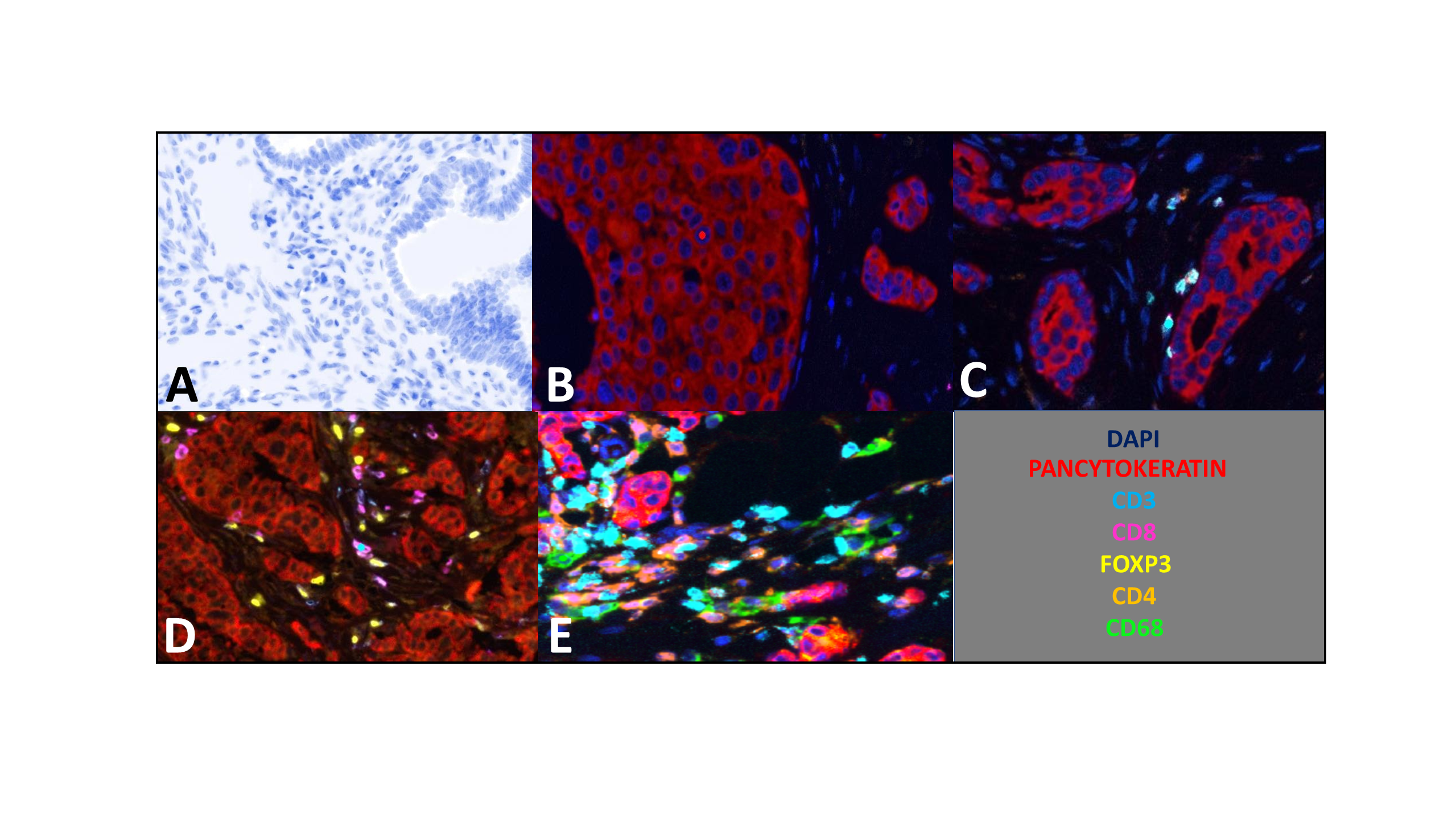

Supplement: Supplementary Figure 3 — qmIF images demonstrating multiplex staining. (A) Dapi/Nuclear single stain (blue) (B) Dapi + pancytokeratin (red) multiplex image, red dot represents training program for cell phenotype (cell assigned as carcinoma based on pancytokeratin staining and nuclear features) (C) Dapi (blue), pancytokeratin (red) and CD3 (cyan) multiplex image (D) pancytokeratin (red), CD8 (magenta), and FOXP3 (yellow) multiplex image (E) Dapi (blue), pancytokeratin (red), CD4 (orange), CD68 (green) multiplex image. [file Image_4.TIF]
